# The Palliative Management of Refractory Cirrhotic Ascites Using the PleurX^**©**^ Catheter

**DOI:** 10.1155/2016/4680543

**Published:** 2016-06-05

**Authors:** Jason Reinglas, Kayvan Amjadi, Bill Petrcich, Franco Momoli, Thomas Shaw-Stiffel

**Affiliations:** ^1^Department of Medicine, Ottawa Hospital Research Institute, University of Ottawa, Ottawa, ON, Canada K1Y 4E9; ^2^Department of Epidemiology and Community Medicine, Ottawa Hospital Research Institute, University of Ottawa, Ottawa, ON, Canada K1Y 4E9

## Abstract

*Background*. Treatment options are limited for patients with refractory cirrhotic ascites (RCA). As such, we assessed the safety and effectiveness of the PleurX catheter for RCA.* Methods*. A retrospective analysis was performed on all patients with RCA who have undergone insertion of the PleurX catheter between 2007 and 2014 at our clinic.* Results*. Thirty-three patients with RCA were included in the study; 4 patients were lost to follow-up. All patients were still symptomatic despite bimonthly large volume paracentesis and were not candidates for TIPS or PV shunt. Technical success was achieved in 100% of patients. The median duration the catheter remained in situ was 117.5 days, with 95% CI of 48–182 days. Drain patency was maintained in 90% of patients. Microorganisms consistent with spontaneous bacterial peritonitis (SBP) from a catheter source were isolated in 38% of patients. The median time to infection was 105 days, with 95% CI of 34–233 days. All patients were treated for SBP successfully with antibiotics.* Conclusion*. Use of the PleurX catheter for the management of RCA carries a high risk for infection when the catheter remains in situ for more than 3 months but has an excellent patency rate and did not result in significant renal injury.

## 1. Background

The development of ascites in the setting of liver cirrhosis heralds a spiraling decline in quality of life and is associated with a 50% mortality within 2 years [1]. Cirrhotic ascites accounts for over 75% of patients who are admitted to hospital with ascites [2]. It is the most common complication of cirrhosis that leads to hospitalization and use of healthcare resources [3].

Refractory ascites, defined as ascites that cannot be mobilized or the early recurrence of ascites which cannot be satisfactorily prevented by medical therapy [4], occurs in approximately 10% of patients with liver cirrhosis [5]. Once ascites becomes refractory to medical therapy, 50% of patients die within 6 months to 1 year [1].

Serial large volume paracentesis (LVP) is currently first-line treatment for refractory ascites and is usually performed as an outpatient every 2 weeks [6]. Potential major complications associated with LVP include bleeding (1%), small bowel perforation (0.4%), and catheter fragmentation into abdominal wall (0.2%) [2, 7, 8]. Although these complications are rare, they are life-threatening.

Transjugular intrahepatic portosystemic stent-shunt (TIPS) may be considered as second-line treatment for refractory ascites in the absence of absolute contraindications for its placement, such as significant heart failure, tricuspid regurgitation, pulmonary artery hypertension, multiple hepatic cysts, systemic infection, and biliary obstruction [9]. A meta-analysis of four randomized controlled trials demonstrated TIPS to be more effective at controlling ascites than serial LVP (70% versus 23%, resp.) but at the cost of greater risk for profound hepatic encephalopathy [10]. Furthermore, many patients with cirrhosis already have several of the aforementioned contraindications to TIPS, thereby limiting its use. In the event a patient is not a candidate for TIPS and has multiple abdominal scars preventing safe serial LVP; a peritoneovenous shunt (PV shunt) performed by a surgeon or interventional radiologist can be considered. However, this procedure is seldom performed due to the lack of survival advantage, excessive complications, and poor long-term patency [11, 12].

Subcutaneous implantation of a battery-powered catheter drainage system, such as the Alpha Pump^©^, is a novel method of managing refractory ascites via directing ascites into the bladder. Limited data exists regarding its safety and efficacy for the management of nonmalignant refractory cirrhotic ascites (RCA). A multicentre case series involving 40 patients revealed successful implantation of the device; however, technical problems with the bladder and peritoneal catheter necessitating intervention were reported in 22% and 12%, respectively. Device failure occurred in 5% of patients and early explants occurred in 32% of patients: 18% were due to infections; the remainder included issues related to dislodgement, bladder hemorrhage, wound dehiscence, and withdrawal of patient consent (reasons not reported) [13].

Indwelling tunneled peritoneal drains, such as the PleurX catheter, for the management of malignant ascites have been demonstrated to improve quality of life and be effective, economical, and safe with low rates of infection [14]. Their use in RCA is controversial due to the lack of data evaluating their efficacy and concerns related to the heightened risk for infection and renal dysfunction in this population [15]. As such, we present a study evaluating the use of the PleurX catheter for the palliative management of RCA.

## 2. Methods

An analysis of data from a prospectively maintained database was performed on all patients with RCA who have undergone insertion of the PleurX catheter between January 2007 and January 2014 at our Chronic Ascites and Recurrent Effusions (CARE) clinic of The Ottawa Hospital. Ethical approval was granted through The Ottawa Hospital Research Institute.

Patients were chosen by a staff Hepatologist for consideration of PleurX insertion if they had failed medical management (i.e., maximum dose of diuretics reached or further diuresis limited due to hypotension and/or renal dysfunction, whilst maintaining a low sodium diet) still symptomatic despite bimonthly LVP and were not considered candidates for alternative methods of treatment (e.g., TIPS and PV shunt). At our institution, TIPS or PV shunt is not performed for refractory cirrhotic ascites; thus patients who are potential candidates for these procedures were referred out to the nearest liver transplant centres for further consideration. In general, patients were not considered candidates for TIPS if they had heart failure, severe tricuspid regurgitation, moderate-to-severe pulmonary artery hypertension (mean pulmonary pressure > 45 mm Hg), multiple hepatic cysts, sepsis, unrelieved biliary obstruction, or difficulties managing hepatic encephalopathy. Patients were not considered candidates for PV shunt if they had end-stage renal failure, significant heart failure, sepsis, uncorrectable coagulopathy, or septation of the peritoneal cavity. Upon the first visit to the CARE clinic, candidacy was reassessed by the interventionist performing the procedure. The patient's file was reviewed, a requisition for bloodwork (CBC, electrolytes, total bilirubin, LFTs, INR, PTT, and albumin) was provided, and informed consent was obtained. To determine patient reliability with regard to follow-up visits, insertion of the PleurX catheter occurred on the second visit 2 weeks later. If the patient did not present to the second appointment, candidacy for insertion of the PleurX catheter was withdrawn. Upon the second visit, preceding bloodwork and patient history were reviewed and baseline ECOG Performance Status and informed consent were obtained prior to PleurX catheter insertion.

The insertion method has been well documented in our recent article [16] and manufacturer's online manual [17]. In summary, all catheters are placed under ultrasound guidance in the largest identified pocket of fluid. The site for insertion is marked and sterilized with a chlorohexidine solution. Under sterile conditions, local anesthesia is achieved with approximately 20 cc of 1% lidocaine. No periprocedural antibiotics are used for our patient population. An 18-gauge needle is then advanced into the peritoneal space, and once ascitic fluid is aspirated, a guide wire is advanced into the peritoneal cavity. Using a number 11 scalpel, a small incision, approximately 7 mm long, is made around the guide wire, ensuring its free mobility. This indicates the entry site for the catheter into the peritoneal cavity. A second incision, 5 mm long, is then made superior and medial to the insertion site. This indicates the exit site for the catheter. The 15.5 French PleurX catheter is then tunneled between the two incisions and retrieved adjacent to the guide wire. The polystyrene cuff is then pulled through the tunnel until it is no longer visible at the exit site. A fascial dilator with a peel-away sheath is then advanced over the guide wire and inserted into the peritoneal cavity. The dilator and the guide wire are then removed and the catheter is advanced into the peritoneal cavity through the peel-away sheath. The sheath is then peeled away while the catheter is simultaneously and completely advanced into the peritoneal cavity, without any kinks that could potentially hinder drainage. Both insertion and exit sites are then sutured with nonabsorbable 2-0 silk suture. A sterile dressing is then applied and covered with a transparent adhesive film. Vital signs are monitored before and after the procedure.

Following insertion of the PleurX catheter and drainage of ascites, a sample of ascitic fluid was sent for cell count and differential, total protein, albumin, gram stain, and culture. First patient follow-ups were scheduled in 1-2 weeks following insertion of the PleurX drain. All of our patients were followed closely by home care nursing services to assist with drainages and dressing changes. The frequency and amount of ascites drained were individualized to each patient based on initial electrolytes, renal function, and blood pressure. After the second visit, the following information was recorded into a database: demographics, etiology of liver disease, preliminary amount of ascites drained from the catheter, adverse events, and technical limitations. For the purposes of this project, additional data on each patient was retrospectively extracted from our electronic database: baseline clinical characteristics, electrolytes, creatinine, ascitic protein and cultures results, adverse events and interventions required, while the PleurX catheter remained in situ, and details involving discontinuation of the catheter or mortality.

Safety was assessed by determining the type and timing of adverse events which were divided into three groups: immediate, occurring less than 24 hours from the procedure; early, occurring 24 hours to 30 days; and late, occurring greater than 30 days from insertion of the PleurX catheter. An adverse event was defined as any complication occurring to the patient which could be related to the catheter. Effectiveness was evaluated by determining technical success and patency and the duration the drain remained in situ. If the patient was lost to follow-up, the duration the drain remained in situ was defined as censored at the patient's last follow-up date. Technical success was defined as successful drain insertion upon the first attempt with subsequent drainage of ascites. Patency was defined as a catheter known to be functioning at death, study end, or resolution of ascites.

Unadjusted risk ratios and confidence intervals were calculated from tabulated frequencies. Testing for associations between the predictors (sex, age, cardiovascular disease, renal disease, hyponatremia, MELD score, Child-Pugh class, and ascitic protein) and the outcomes (spontaneous bacterial peritonitis and adverse events) was performed using chi-square and Fisher's exact tests. A high MELD score was defined as ≥15 given that the 3-month mortality progressively accelerates beyond this point [18]. Paired* t*-tests were used to assess for significant changes in electrolytes and renal function following the placement of the PleurX catheter. Kaplan-Meier curves were fit to censored follow-up data to derive median duration of first adverse event, spontaneous bacterial peritonitis, and duration the drain remained in situ. Multivariate estimates of relative risk were obtained using binomial generalized linear models.

## 3. Results

Over a 7-year period, 33 patients with RCA were chosen for PleurX catheter insertion and followed until withdrawal of the catheter or death. All patients failed medical management and were still symptomatic despite bimonthly paracentesis. Twenty-five (75.8%) patients were not candidates for liver transplant due to alcohol abuse (33.3%), heart failure and/or chronic kidney disease (30.3%), age (9.1%), and unknown reasons in 1 patient due to missing documentation. The remainder of the cohort included: 6 patients (18.2%) on the liver transplant wait list, 1 patient who died prior to obtaining a final decision from the transplant centre regarding their candidacy, and 1 patient who refused liver transplant. The majority of patients (54.5%) were not considered candidates for TIPS due to difficulties controlling hepatic encephalopathy. The remaining patients were not considered for TIPS due to heart failure and/or chronic kidney disease (36.4%) and unknown reasons in 3 (9.1%) patients. Four patients were lost to follow-up after the PleurX catheter was inserted. The study group, described in Table 1, included 19 males and 14 females (age range: 44–87; mean: 62 years). The etiology of cirrhosis was alcohol in the majority of patients with an average MELD score and ECOG Performance Status of 17 and 3.12, respectively. Most patients were hyponatremic and had underlying chronic kidney disease. During the follow-up period, nine deaths were recorded: 5 patients died secondary to respiratory failure in the context of hepatic encephalopathy, 1 patient died secondary to a variceal bleed, and 3 patients died outside hospital for unknown reasons. One patient still had their PleurX catheter in situ at the time of chart review.

Technical success was achieved in 100% of patients without any immediate complications (i.e., bleeding, bowel perforation, needle-catheter fragmentation, or procedure-related deaths). The average amount of ascites drained upon insertion of the PleurX catheter was 8.53 litres (range: 3–18.5). The average ascitic protein concentration was 18 g/L, and four patients had less than 10 g/L. Most patients had 2 L drained three times per week (range: 2 L per week to 1 L per day). Overall, the median duration the catheter remained in situ was 117.5 days, with 95% confidence interval (CI) of 48–182 days. The median duration the catheter remained in situ was longer in patients with a MELD score of less than 15 as compared to those with a MELD score greater than or equal to 15 (123 days, 95% CI, 34–240 days versus 95 days, and 95% CI, 34–187 days, resp.), though not statistically significant. Drain patency was maintained in 90% of study subjects.

**Figure 1:**
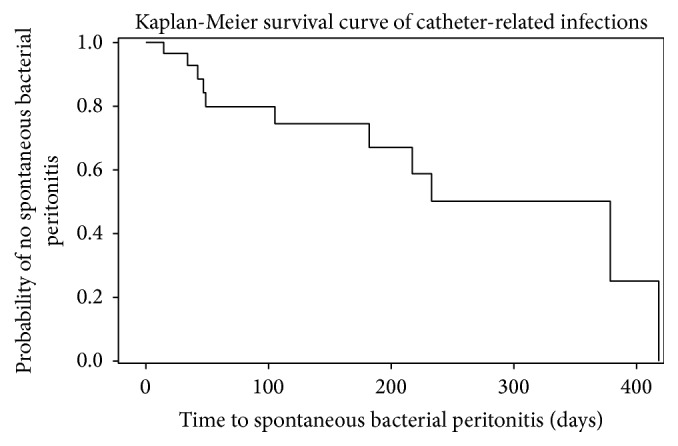
Kaplan-Meier curve displaying the interval between drain insertion and spontaneous bacterial peritonitis secondary to microorganisms consistent with skin flora (i.e.,* Staphylococcus*,* Pseudomonas*,* Bacillus* species,* Coryneform*, and* Acinetobacter*).

Overall, adverse events occurred in 59% (*n* = 17) of patients (see Table 2). The median time to first adverse event was 182 days, with 95% CI of 49–258 days. In multivariate analysis after controlling for age, gender, and ECOG Performance Status, patients with Child-Pugh class B liver disease were not more likely to encounter an adverse event as compared to Child-Pugh class C patients (RR: 2.2, 95% CI: 0.94–5.01; *p* = 0.07). Infections were the most common complication; over the study period 48% (*n* = 14) of patients had positive peritoneal fluid cultures. Typical spontaneous bacterial peritonitis (SBP) enteric microorganisms (i.e.,* Escherichia coli*,* Enterococcus*,* Klebsiella*, and* Streptococcus*) were isolated in 21% (*n* = 6) of patients. Microorganisms consistent with SBP from a catheter source (i.e.,* Staphylococcus*,* Pseudomonas*,* Bacillus* species,* Coryneform*, and* Acinetobacter*) were isolated in 38% (*n* = 11) of patients. The median time to infection with microorganisms consistent with a catheter source was 105 days, with 95% CI of 34–233 days (see Figure 1).  Table 3 displays the relative risk ratios of the predictors associated with the outcomes for SBP from a catheter source and time to first adverse event. A MELD score of greater than or equal to 15 was significantly associated with being less likely to contract SBP from the catheter (RR: 0.3, 95% CI: 0.09–0.84; *p* = 0.02). However, patients with Child-Pugh class B were not more likely to contract SBP from the catheter as compared to Child-Pugh class C (RR: 6.5, 95% CI: 0.96–43.72; *p* = 0.06). Following multivariate analysis, both MELD score and CPS class were not associated with SBP. SBP caused by typical enteric microorganisms was not significantly associated with any of the predictors. Two patients grew out typical enteric microorganisms on one occasion and microorganisms consistent with a catheter source on another occasion. One patient grew out both coagulase negative* Staphylococcus* and* Peptostreptococcus* in 1 culture, and 1 patient had 4 episodes of SBP secondary to typical enteric microorganisms. Three patients developed localized cellulitis at the catheter site. Six patients had their catheters removed for source control, and all were treated successfully with antibiotics.

Minor early complications (i.e., 24 hours to 30 days) occurred in 5 patients. Three patients with significant anasarca and skin edema around the catheter site developed a leak between the catheter's polyester cuff and the skin. The leak resolved spontaneously in 2 patients with subsequent drainages, and 1 patient required the application of a dermal adhesive and ultimately had their catheter removed due to persistent leakage and skin site irritation. Three patients with chronic renal disease developed prerenal azotemia, the frequency of drainages were reduced, and their renal function returned back to baseline. One catheter became displaced and was subsequently removed. One patient developed a hematoma which resolved spontaneously. From time of the insertion to first follow-up, patients were on average significantly (*p* < 0.05) though mildly more hyponatremic (−2 mmol/L ± 4 mmol/L) and hyperkalemic (0.4 mmol/L ± 0.8 mmol/L), with subtle increases in their creatinine (12 *μ*mol/L ± 29 *μ*mol/L).

Minor late complications (i.e., greater than 30 days) occurred in 8 patients. Four patients developed leaks, three resolved spontaneously, and one required the application of sutures for control. Three drains became blocked; two patients had their drains successfully replaced; one patient's drain was successfully unblocked with tPA.

## 4. Discussion

Serial LVP is currently the mainstay of treatment for patients with RCA who are not candidates for TIPS or PV shunt [1]. Rarely, this procedure is required more often than every 2 weeks and most patients with RCA can likely be successfully managed by this modality. However, our patients chosen for PleurX catheter insertion were still severely symptomatic with some necessitating frequent Emergency Department visits for emergent paracentesis, despite bimonthly paracentesis and maximal dosing of diuretics. Although infrequent, serial LVP does carry significant risks. In one large single-centre prospective study, De Gottardi et al. evaluated complications associated with 515 paracenteses in 171 patients with liver cirrhosis [8]. Major complications, which resulted in the death of two patients, occurred in 1.8% of the cohort. Furthermore, 134 technical problems occurred in 29 patients, and 5% of patients developed issues with puncture site leakage following the procedure. In contrast, our study was consistent with a review of 9 studies evaluating the PleurX catheter for the management of malignant ascites which demonstrated excellent technical success rates without any major complications involving bleeding, bowel perforation, or catheter fragmentation [14].

The longevity of the catheter as determined by drain patency and duration in situ was greater in our study than what has been previously reported in studies evaluating the PleurX catheter in patients with malignant ascites. To our knowledge, the longest duration found in the literature for patients with malignant ascites was documented to be an average of 113 days (95% confidence interval: 70–157 days) in a prospective study by Tapping et al. in 28 patients with mainly gastrointestinal and genitourinary primary tumours [19]. The shorter duration the catheter remained in situ in studies evaluating patients with malignant ascites was likely secondary to worse prognoses. Patency rates in the literature also varied amongst patients with malignant ascites and were between 67.5 and 85% [19–21], as compared to our patency rate of 90%. This difference could be accounted for by the greater concentration of protein in malignant ascites as compared to cirrhotic ascites, resulting in a more vicious fluid causing a greater number of drain blockages.

Postparacentesis circulatory dysfunction is another complication of serial LVP, which becomes most significant when the volume extracted is greater than 5 litres in one session [22]. Tunneled indwelling peritoneal catheters, such as the PleurX drain, carry the potential to help reduce the incidence of postparacentesis circulatory dysfunction by enabling more frequent but less extensive drainages titrated to the patients symptomology, blood pressure, and renal function. As supported by this small study, renal function was grossly preserved in patients undergoing more frequent but smaller volume drainages. At present, there are limited studies available assessing the complications associated with placement of a PleurX drain in patients with RCA likely secondary to concerns related to infection [23].

Infection is a common complication of liver disease which has been attributed mainly to dysfunctional neutrophilic and reticuloendothelial systems [24]. As a result of decreased opsonic activity secondary to low ascitic protein concentrations, ascites can provide a favorable environment for bacteria to flourish [24]. Despite this, Imler et al. reviewed 26 patients with advanced liver disease who underwent PleurX catheter insertion for the management of hepatic hydrothorax or ascites and found that 2 patients developed infections (*Candida glabrata* peritonitis and* E. coli* sepsis) after 30 days, concluding a low incidence of infections [23]. Similarly, Chalhoub et al. used the PleurX catheter for the management of hepatic hydrothorax in 8 patients and found that 1 patient developed an exit site infection towards the end of the study [25]. In contrast, Nadir and Van Thiel inserted 38 peritoneal drainage catheters in 30 patients with cirrhosis for durations ranging from 1 to 10 days and found that patients were more likely to develop positive ascitic cultures if the catheters were left in for greater than 2 days [26]. However, the study performed by Nadir and Van Thiel did not use tunneled catheters with polyester cuffs, as is present on the PleurX catheter. The polyester cuff stimulates an inflammatory reaction at the cuff-skin interface promoting granulation and tissue ingrowth onto the cuff, thereby enhancing the seal and reducing the possibility for infection [14]. Furthermore, tunneled catheters are well known for their lower risk of infection over nontunneled catheters [27]. Despite the additional barriers to infection provided by the PleurX catheter, we observed a very high incidence of infection. As compared to the incidence of infection after PleurX insertion for malignant ascites (3–18%) [19–21], 38% of patients in our group developed positive ascitic cultures with microorganisms consistent with skin flora attributed to the catheter and 6 patients required removal of the catheter for source control. However, the median time to infection in our group was considerably longer than those found in the aforementioned studies. In comparison, only 1 patient developed a catheter related ascitic fluid infection in our cohort after 30 days.

A relationship between less severe liver disease, as determined by MELD, and greater risk for SBP was initially supported in univariate analysis but lost in multivariate analysis. The lack of relationship between severity of liver disease and SBP has been previously reported in the literature. Haddad et al. performed a prospective study evaluating the rate of SBP in 148 patients undergoing serial LVP; following 854 paracentesis procedures, MELD was not associated with the development of SBP [28].

There were several limitations to this study. Given that refractory ascites constitutes a small proportion of patients with liver disease and that an even smaller proportion fails conservative management, despite collecting patient data over 7 years, the sample population in this study is small. The small sample size is reflected in the large confidence intervals, thus interpreting the statistical analyses is challenging due to the low power of the study. As there was no comparison group, we were limited in comparing our findings to other studies. Our institution has a specific centre which manages ascites; as a result, our protocols likely differ considerably from other institutions. Regarding the patients who were lost to follow-up, it is unclear whether any morbidity or mortality was associated with the PleurX catheter. Although they were censored from the analyses, given the small sample size, their data would have been valuable. Although these limitations are significant, the strength of this paper lies in the descriptive data. To our knowledge, this study has the most extensively monitored follow-up period evaluating the use of the PleurX catheter for the management of RCA, which was greater than 1 year for some patients. It is sensible to assume that larger prospective studies will not be able to be performed in a reasonable amount of time to address the statistical power of this study.

## 5. Conclusion

Use of the PleurX catheter for the management of RCA carries a high risk for infection when the catheter remains in situ for greater than 3 months but may prevent significant renal injury encountered in LVP and has an excellent patency rate. In the small subgroup of patients with refractory cirrhotic ascites who are still symptomatic or are unable to tolerate bimonthly large volume paracentesis and are not candidates for TIPS or PV shunt, the use of the PleurX catheter is a potential treatment option. Discussions with patients should include balancing the high risk for infection and consideration of prophylactic antibiotics against the potential preservation of renal function and improvement in one's quality of life.

## Figures and Tables

**Table 1:** Patient demographics and baseline clinical characteristics.

Total number of patients	33
Male gender	19 (57.5%)
Mean age	62 (range 44–87)

*Etiology of liver cirrhosis*	
Alcohol	12 (36.4%)
Hepatitis C	7 (21.2%)
Nonalcoholic steatohepatitis (NASH)	7 (21.2%)
Alcohol and hepatitis C	4 (12.1%)
Cardiogenic	3 (9.1%)

*ECOG performance status*	
2	4 (12.1%)
3	21 (63.6%)
4	8 (24.2%)
Renal disease^*∗*^	23 (69.7%)
Cardiovascular disease^i^	8 (24.2%)
Hyponatremia (<135 mmol/L)	20 (60.6%)
Ascitic fluid protein <10	4 (12.1%)

*Mean model for end-stage liver disease (MELD) score* ^€^	17 (range 8–31)
Number of patients with MELD ≥ 15	18 (54.5%)
Number of patients with MELD < 15	13 (39.4%)

*Child-Pugh class* ^€^	
A	0
B	19 (57.5%)
C	12 (36.4%)

^*∗*^Chronic renal disease was determined to be present if the patient's eGFR was less than 60 mL/min/1.73 m^2^ on baseline bloodwork.

^i^Cardiovascular disease was determined to be present if the patient reported a history of such or if there was evidence of coronary artery disease, ischemic cardiomyopathy, cerebrovascular disease, or peripheral artery disease on the patient's online medical record.

^€^MELD and Child-Pugh class could not be reliably calculated for two patients due to their use of warfarin.

**Table 2:** Adverse events following PleurX catheter insertion (*N* = 17).

Adverse event	*n* (%)
Ascites outflow around the catheter site	7 (41.2)
Hematoma	1 (5.8)
Catheter obstructed	3 (17.6)
Catheter displaced	1 (5.8)
Cellulitis	3 (17.6)
Spontaneous bacterial peritonitis (enteric^‡^)	6 (35.3)
Spontaneous bacterial peritonitis (catheter-associated^†^)	11 (64.7)
Prerenal azotemia	3 (17.6)

^‡^Enteric, includes typical microorganisms known to be associated with spontaneous bacterial peritonitis in liver cirrhosis (i.e., *Escherichia coli*, *Enterococcus*, *Klebsiella*, and *Streptococcus*).

^†^Catheter-associated, includes microorganisms consistent with skin flora (i.e., *Staphylococcus*, *Pseudomonas*, *Bacillus* species, *Coryneform*, and *Acinetobacter*).

**Table 3:** Relative risks (RR) for variables related to catheter-associated SBP and adverse events^i^.

Variable	Catheter-associated SBP^‡^		Adverse events	
	RR	95% CI^†^	RR	95% CI
Age ≥ 65 years	1.4	0.54, 3.42	0.9	0.49, 1.83
Male gender	0.9	0.33, 2.14	1.2	0.62, 2.40
Cardiovascular disease	1.8	0.70, 4.36	1.4	0.74, 2.52
Chronic kidney disease	1.0	0.36, 2.90	1.2	0.54, 2.57
ECOG^!^ ≥ 3	1.6	0.27, 9.33	1.2	0.41, 3.24
Hyponatremia^¥^	2.0	0.65, 6.26	1.2	0.62, 2.34
Ascitic protein > 10 g/L	1.8	0.31, 10.55	1.3	0.47, 3.63
MELD^§^≥ 15	0.3^•^	0.09, 0.84	0.6	0.34, 1.14
Child-Pugh class B	6.5	0.96, 43.72	2.0	0.86, 4.57

^i^Adverse events included catheter obstruction or displacement, infections, prerenal azotemia, hematoma, or ascites outflow around the catheter site.

^‡^SBP, spontaneous bacterial peritonitis.

^†^CI, confidence interval.

^!^Eastern cooperative oncology group performance status score.

^¥^Serum sodium < 135 mmol/L.

^§^Model for end-stage liver disease score.

^•^ *p* value ≤ 0.05.
